# A Multimodal Emotion Detection System during Human-Robot Interaction

**DOI:** 10.3390/s131115549

**Published:** 2013-11-14

**Authors:** Fernando Alonso-Martín, María Malfaz, João Sequeira, Javier F. Gorostiza, Miguel A. Salichs

**Affiliations:** 1 Robotics Lab, Universidad Carlos III de Madrid, Av. de la Universidad 30, Leganés, Madrid 28911, Spain; E-Mails: mmalfaz@ing.uc3m.es (M.M.); jgorosti@ing.uc3m.es (J.F.G.); salichs@ing.uc3m.es (M.A.S.); 2 Institute for Systems and Robotics (ISR), North Tower, Av.Rovisco Pais 1, Lisbon, 1049-001, Portugal; E-Mail: jseq@isr.ist.utl.pt

**Keywords:** emotion recognition, affective computing, human, robot interaction, dialog systems, FACS

## Abstract

In this paper, a multimodal user-emotion detection system for social robots is presented. This system is intended to be used during human–robot interaction, and it is integrated as part of the overall interaction system of the robot: the Robotics Dialog System (RDS). Two modes are used to detect emotions: the voice and face expression analysis. In order to analyze the voice of the user, a new component has been developed: Gender and Emotion Voice Analysis (GEVA), which is written using the Chuck language. For emotion detection in facial expressions, the system, Gender and Emotion Facial Analysis (GEFA), has been also developed. This last system integrates two third-party solutions: Sophisticated High-speed Object Recognition Engine (SHORE) and Computer Expression Recognition Toolbox (CERT). Once these new components (GEVA and GEFA) give their results, a decision rule is applied in order to combine the information given by both of them. The result of this rule, the detected emotion, is integrated into the dialog system through communicative acts. Hence, each communicative act gives, among other things, the detected emotion of the user to the RDS so it can adapt its strategy in order to get a greater satisfaction degree during the human–robot dialog. Each of the new components, GEVA and GEFA, can also be used individually. Moreover, they are integrated with the robotic control platform ROS (Robot Operating System). Several experiments with real users were performed to determine the accuracy of each component and to set the final decision rule. The results obtained from applying this decision rule in these experiments show a high success rate in automatic user emotion recognition, improving the results given by the two information channels (audio and visual) separately.

## 1. Introduction

In robotics, the main objective of automatic dialog systems is to get a natural interaction between the human and the robot, similar to the one among humans. This would help to eliminate the need of artifacts, such as keyboards and mouses, favoring more intuitive and more suitable ways of interaction for non-expert and/or disabled users. Nevertheless, at present, the dialog systems developed for human–robot interaction (HRI) are not comparable to human dialog. This is due, among other reasons, to the fact that the interaction among humans involves not only the interchange of explicit information, such as the literal sentence transmitted by voice, but also the implicit one, such as the information about the emotional state of the interlocutor. Therefore, including this information in dialog systems would improve their performance.

This kind of system, which includes this emotional information, is related to a recent research area known as “affective computing”, introduced by Picard [1]. This area comprises emotion recognition, management and its generation.

**Figure 1.**
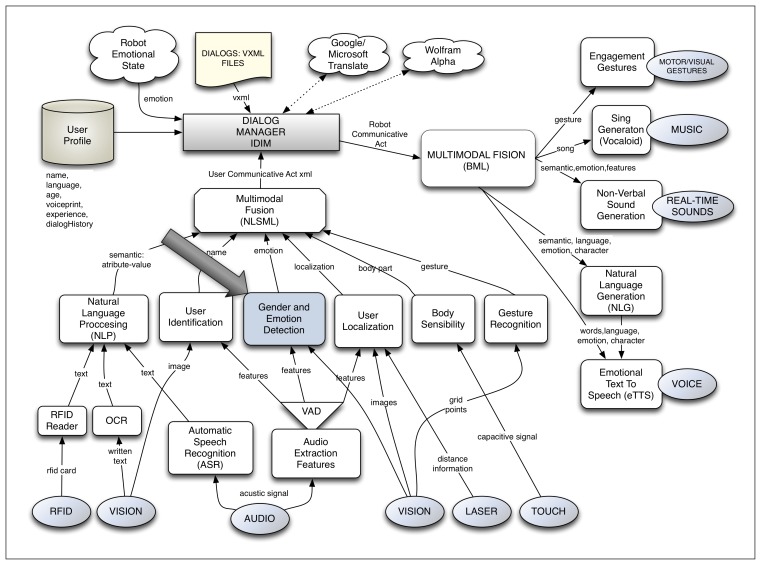
The multimodal interaction system Robotics Dialog System (RDS).

The work presented in this paper is developed in a complete multimodal dialog system applied to HRI. In this interaction system, called the Robotics Dialog System (RDS) [2,3], user emotion detection is considered as another module. A general view of this dialog system can be observed in Figure 1. The modules related to the sensitive part of the interaction are situated in the lower part; meanwhile, the expressive ones can be found at the right part of the figure. The dialog manager is placed in the central part of the figure and manages the dialog flow. Finally, the multimodal fusion module, which is in charge of fusing together the information given by each sensitive module in communicative acts (CAs), is placed between the dialog manager and the sensitive modules. Each of these CAs corresponds to a dialog turn and contains the information that the user wants to interchange with the robot. It is in these CAs where the information about the user emotion needs to be included.

Let us see an example of the information contained in a CA. In this case, the user wants the robot to turn the TV on:

1. text = “I want to watch television”
2. action = turnOn
3. object = television
4. user = Fernando
5. location = 30,10,4
6. bodyPose = standUp
7. touched = nothing
8. emotion = neutral

As observed, the information included in the CA is: the semantic values associated with the transmitted sentence (1,2,3); the name of the user (4); the location in relation to the robot (5); the pose of the user body (6); the touched part of the robot body (7); and the detected user emotion (8).

The inclusion of a specific module for emotion detection in the RDS intends to improve the HRI by augmenting its naturalness and “engagement” [4]. Using the detected user emotion, the dialog could be customized according to a certain user and to a specific communicative situation. Moreover, some misunderstandings between the user and the robot, which could result in boredom, *etc.*, could be prevented. In this same line, the robot, by directing a question to the user, could confirm the emotion detected and even take the initiative to change the emotional state of the user.

In this work, the proposed multimodal emotion detection system is described and implemented. This system uses two information channels or modes: audio and visual. As will be shown, the system is composed of two components, Gender and Emotion Voice Analysis (GEVA) and Gender and Emotion Facial Analysis (GEFA), which analyze the information received by both channels to determine the emotion of the user. GEVA is in charge of analyzing the audio signal, and GEFA uses two third-party software packages to obtain the detected emotion through the facial expression analysis. The outputs given by both components are combined using a decision rule to finally determine the main detected user emotion.

As already said, this user emotion detection system is implemented in an overall multimodal interaction system (RDS). In this sense, other works have presented emotion detection systems, but none of them have been integrated in a system that manages the interaction between users and social robots. In addition, the component, GEVA, has also been developed for voice activity detection.

The experimental part has two phases: first, the success rates of both components (GEVA and GEFA) are calculated separately; second, the success rate of the whole system is obtained using both modules (and the information received by the audio and visual channels) and the decision rule.

In the first part, the experiments were carried out with about 70 users who interacted with the system expressing emotions in a natural and also in a faked way. These experiments helped to define the decision rule of the whole system. Finally, other experiments with real users, who were asked to fake their emotions, prove the improvement of the performance of the multimodal system in recognizing the user emotion.

This paper is structured as follows. In Section 2, some issues related to the automatic emotion detection process and the point of view of other researchers are presented. Section 3 describes the proposed multimodal user emotion detection system. Next, in Section 4, the developed component, GEVA, for emotion detection using voice analysis is described. Section 5 introduces the emotion detection system through facial expression analysis, GEFA. In Section 6, the whole system, which includes GEVA, GEFA and the decision rule, is detailed. Next, the experiments and their results are presented in Sections 7 and 8, respectively. Finally, the conclusions are presented in Section 9.

## 2. Related Issues: State-of-the-Art

Regarding automatic emotion detection, some issues must be considered. In this section, these issues, and the point of view of other researchers, are described.

### 2.1. What Emotions Must Be Detected?

According to the literature, in order to classify or tag an emotion, there are mainly two approximations.

The first one consists of a discrete set of basic emotions and, in some cases, of secondary emotions generated as combinations of the first ones [5]. In this sense, according to Ekman [6], a set of six basic emotions can be defined: joy, disgust, anger, fear, surprise and sadness.

The second approximation consists of measuring and contextualizing emotions according to different dimensions. Therefore, each emotion can be viewed as points or areas in the space defined by these dimensions. In the works by Plutchik [7] and Cowie [8], two dimensions are established: activation and evaluation. Activation is understood as the predisposition of the person to execute an action according to his emotional state. Evaluation reflects the global appraisal of the positive or negative feeling associated with the emotional state. Another approach is the one presented by Bradley [9,10], who uses an arousal-pleasure model. The first dimension explains the desire and the second one, the physiological activity related to the affective state.

### 2.2. What Channels Could Be Used for Emotion Detection?

In the literature, there are some works related to the automatic emotion detection process. Many of them are related to facial expression analysis through artificial vision [11–13] and to user voice analysis to detect the emotion of the interlocutor [14–19]. Moreover, just a few describe multimodal systems (using several input channels) for detecting emotions [20–22], although none of them is implemented in a dialog system for HRI.

De Silva [23] stated that, for humans, some emotions are better identified by voice, such as sadness and fear, but others are better detected through facial expression analysis, such as happiness and anger. Besides, Chen [24] showed that these two modalities give complementary information, since the performance of the system increases when both of them are considered together. This same idea seems to be also corroborated by Tu [25], who obtained a success rate of 60% when using the voice channel and of 57% in the case of using the visual one, but, considering just one multimodal classifier, the success rate increases up to 72%. Moreover, a complete survey of the use of the audio and visual channels for detecting spontaneous emotions is presented in [26].

Yoshitomi [27] complements the facial features extraction with the addition of a non-intrusive channel of information: the image obtained from an infrared camera. In this work, there is a classifier for each of the three channels, and then, the information given by each of them is fused together. Again, they obtained better results when using the whole system in comparison with the results obtained with each channel separately. The success rate is 60% using the audio channel, 56% with the visual one, 48% with the infrared image and 85% when using the whole system.

On the other hand, the information received by the audio and vision channels could be also used in other ways to detect the user emotion, such as making an emotional analysis of the information transmitted verbally, using natural language processing techniques, as shown in [28–30], analyzing previous dialogs with the user and analyzing his body pose, too [31,32].

Finally, it is worth mentioning that during the last two decades, several studies have been focused on emotion detection using brain-machine interfaces. They are based on the analysis of the neural activity of the users [33,34]. The most used technique is electroencephalography (EEG) signal analysis. These works explain how to work with these signals and how to extract information using the Discrete Wavelet Transform (DWT)-this is the transform that allows the processing of the non-stationary signals obtained from the EEG. In these cases, the success rate is between 55% and 76%.

### 2.3. At Which Level Must the Information Received from Each Channel be Fused?

In general, there are two phases in the emotion detection process: the features extraction phase and the classification phase. Since we consider a multimodal system, it is necessary to determine when to fuse the information received from each channel.

On the one hand, there is the possibility of having a unique classifier that receives the extracted features by each channel as inputs. Using that information, the classifier makes the decision about the user emotion using some kind of algorithm. In this case, it is said that the fusion is made at the “features extraction level”.

On the other hand, it can happen that each channel has a classifier that determines the detected emotion using just the information perceived by this channel. In this case, a decision rule is necessary to determine the final principal emotion. This rule must consider the emotion perceived by each channel and its confidence. In this case, the information fusion is made at the “decision level”. In this sense, Mehrabian [35,36] gives the following formula to establish the “weights” given to each channel during the emotion detection: sentence meaning (semantic) 7%, intonation 38% and face 55% (the well-known “7%–38%–55% rule”).

**Figure 2.**
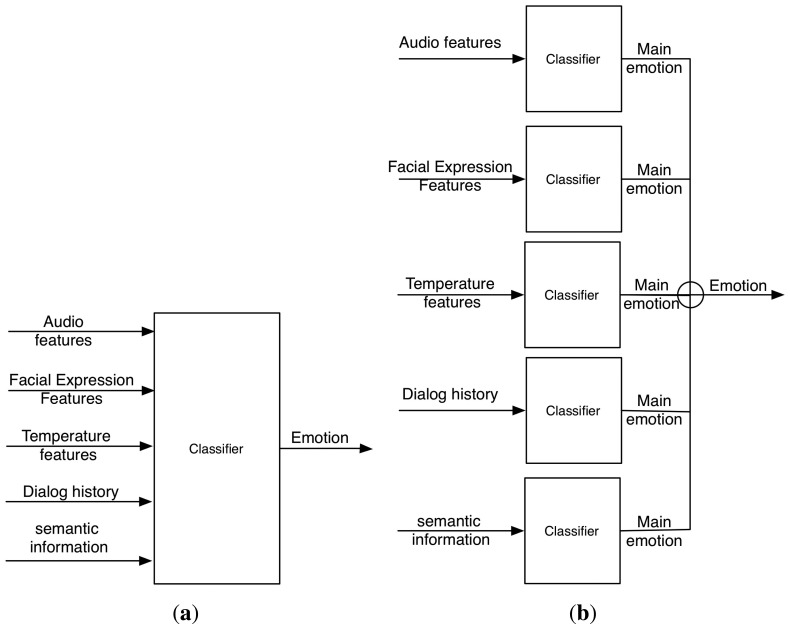
Two kinds of fusion levels: decision and feature extraction level. (**a**) A unique classifier (fusion at the feature extraction level); (**b**) one classifier for each channel (fusion at the decision level).

In Figure 2, both approaches are illustrated using several sensitive channels.

Truong [37] presents a summary of several experiments by other authors, mainly Chen, Sebe and DaSilva. The experiment carried out by Chen [38] showed that the success rate using the audio channel is 75%; the one for the visual channel is 69%, and the success rate obtained for their combination at the feature level (one classifier for both channels) reaches 97%. In the same line, Sebe [39] obtained that the following success rates are 45% (audio), 56% (visual) and 89% when the fusion of both channels is also made at the feature extraction level. On the contrary, De Silva [23] makes the fusion at the decision level and claims to have a 72% success rate. These works and the others presented in [37] seem to prove the following two points: first, that the fusion of the information from several channels improves the success rate; and second, that the fusion made at the feature extraction level obtains higher successful results. Nevertheless, Truong comments that these comparisons are not very rigorous, since, among other things, the emotion sets considered in these works are different.

### 2.4. Types of Training

Once the decision about having one classifier for each channel or just a single classifier is made, then it is necessary to determine if these must be trained for each user, for each group of users (for example, if they have the same cultural characteristics, such as the language) or for any kind of user. Besides, it must be decided if the training is made during real interactions with the system (*online*) or if it is made in previous sessions (*offline*). On the other hand, it is also necessary to decide if the training is made using unnatural or natural expressions of the emotions.

### 2.5. Real Applications

The use of automatic user emotion detection systems is very extended in several fields. For example, Yildirin [40] uses emotion detection to adapt the dialog of a computer game to the user state. He considers three states: neutral, polite and frustrated. Recently, the second version of the Microsoft Kinect incorporates facial expression detection to improve the adaptation of the game to the player [41].

Emotion detection is also being used for medical applications. For example, Toledo-Ronen [42] monitors the emotional state of patients with dementia to try to detect depression cases. Komlosi [43] extended the previous application to schizophrenic patients, and in [44], it is used to monitor autistic patients.

Another use can be found in call centers, where emotion detection can be used as part of a decision support system for prioritizing voice messages and for assigning the proper agent to answer the message [45]. Moreover, Inanoglu, in [46], prioritizes the voice messages according to the expressed emotion in each of them.

A different application is presented by the Spanish company, Biloop [47], who developed its own software/hardware for detecting and classifying babies'emotions by analyzing the babies' sounds.

## 3. Characteristics of the Proposed Multimodal Emotion Detection System

**Figure 3.**
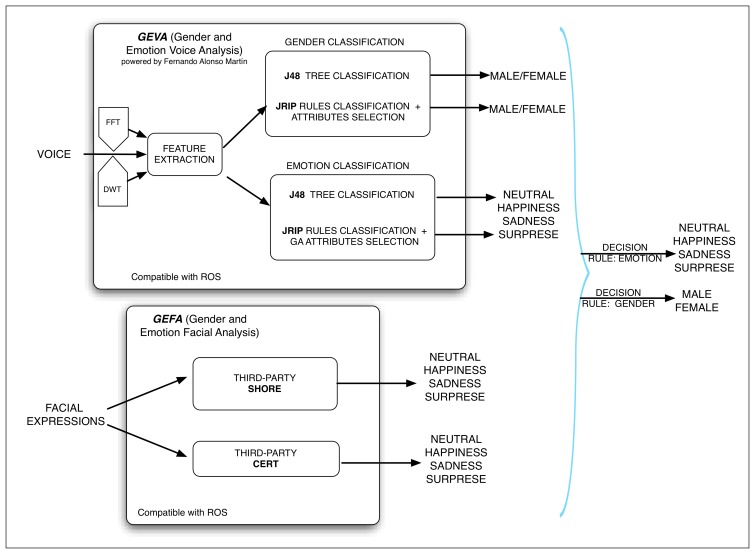
Multimodal emotion detection system.

As already explained, in this work, the multimodal emotion detection system is implemented in the general interaction system (RDS) of a social robot to improve the HRI. In this section, the developed system is described. A general scheme of the system can be viewed in Figure 3. Next, each of the related issues enumerated in the previous section is analyzed.

### 3.1. Representation of the Detected Emotions

As stated in Section 2.1, there are two approximations to classify the detected emotions: the discrete approach and the use of an affective space. In our approach, we have adopted the first option for the following reasons:

- To simplify the implementation of new interaction dialogs: Since the dialog system uses the perceived user emotion and this dialog is specified by an XML file [48] (which follows the VoiceXML [49] standard and is interpreted by the dialog manager, that is based on the slot filling tool [50], it is much easier to write sentences, such as: “*if the user is happy, I offer to play a game*” instead of: “*if the activation level is ⩾50 and the evaluation level is ⩾90, then I offer to play a game*”.
- To simplify the unification of information given by the different channels used to detect the user emotion.

The selected set of emotions is the following: *neutral, happiness, sadness* and *surprise*. The reasons for selecting these four emotions are the following:

- A small set of emotions is required, since the success rate in emotion detection decreases as the number of emotions increases.
- For the HRI, these emotions have been considered to be the most relevant ones in order to personalize the dialog.
- These are emotions that are easily distinguished by the different channels used. Some of the emotions proposed by Ekman have not been considered (fear, disgust and anger), since the considered visual tools have difficulties in detecting them.

Moreover, neutral emotion has been considered, since, as other authors say [51], frequently, the percentage of neutral talk is much higher than the emotional one. Therefore, in the automatic emotion detection area, the neutral state is considered as another emotion.

### 3.2. Channels Used for Detecting Emotions

As humans do, several channels can be used to perceive and detect the emotion of the interlocutor during a conversation. In this work, the channels used are the audio (user voice analysis) and the visual (facial expressions analysis) ones. In order to carry out the user voice analysis, the component, GEVA (Gender and Emotion Voice Analysis), has been developed (although out of the scope of the paper, the software developed is also able to detect the user gender). On the other hand, to analyze the facial expressions of the user, the system uses the component, GEFA (Gender and Emotion Facial Analysis). This component is composed of two third-party software packages (Sophisticated High-speed Object Recognition Engine (SHORE) and Computer Expression Recognition Toolbox (CERT).

At the moment, the use of invasive techniques has been discarded, since the user satisfaction during the interaction could be decreased.

### 3.3. Level at Which the Information Given by Each Channel is Fused

As described in Section 2.3, there are two alternatives regarding the fusion of the information received by different channels: fusion at the decision level (one classifier for each channel and a decision rule) and fusion at the feature extraction level (a unique classifier).

It is quite difficult to compare both approaches, since, as previously shown in Section 2.3, these works have different emotional sets, users, audio and image quality, *etc*. In this work, we have adopted the first approximation: fusion at the decision level. Therefore, there is a classifier for each channel (audio and visual) and, finally, a decision rule, which determines the principal user emotion according to the outputs of both classifiers. The main reasons for selecting this option are the following:

1. Historic: at the beginning of our research, we developed a classifier for the voice analysis, and later, the visual channel was added.
2. Easiness of development and evaluation: it is easier to “debug” each system separately than a combined system, since there are less factors involved. Besides, if a high success rate is obtained for each channel, a combined system would also have good results.
3. Easiness of integration: since two third-party software packages are used for the visual channel, it is not possible to obtain the facial expression features. Therefore, we could not use a single classifier.

### 3.4. Type of Training

The construction of the classifier needs a learning or training process made by using emotion-tagged voice samples. In this work, the training of the classifiers used for the audio channel has been done offline using previously obtained examples. Its working is universal, in the sense that there is no need for training for each user or culture (language). Moreover, unnatural and natural emotional expressions were used for the training phase.

The natural (spontaneous) expressions were obtained from the analysis of natural conversations found on the Internet. On the other hand, the unnatural ones were obtained from interviews with users, who were asked to fake a certain emotion, and from TV shows and films.

The software packages used for the facial expression analysis are already trained.

## 4. The Audio-Based Emotion Detection Component: GEVA

In the literature, there is evidence about how the change of the interlocutor emotion affects voice tone [52]. These changes are reflected in prosodic variations of the voice. Nevertheless, users rarely show a “pure emotion” in the sense that they almost never express happiness or sadness without being mixed with other emotions.

In order to detect the user emotion using the audio channel, two phases are needed: the first one is the voice features extraction, and the second one is the construction of the classifier, the output of which is the perceived emotion. In order to build this classifier, it is necessary to know the considered features, the set of emotions and the set of training locutions (denominated *corpus*).

### 4.1. Voice Features Extraction

There are several open sound feature extraction systems. In this work, we have experimented with “OpenSMILE” [53] and with another version that includes the emotion classifier, “OpenEAR” [54]. Another voice features extraction system is “Praat” [55], but in our opinion, it is not easy to use for an online extraction. It is more suitable for working with prerecorded sounds in a graphical environment. Verbio Speech Analytics [56] also gives solutions for analyzing the audio and detecting certain emotions (anger, joy and neutral).

In this work, for the voice features extraction, we have decided to use the language, Chuck [57]. This language is intended to be used for analyzing sound waves and for generating non-verbal sounds. It has been selected for its capacity for working online and for its simplicity and elegance in relation to the programming, using its own audio analyzers. Currently, Chuck is being used by millions of people through apps for Android and Apple iOS, under the company SMULE. Its versatility and power for synthesis and analysis can be tested using the tool, Wekinator [58].

A complete and independent module in charge of the feature extraction has been developed. This module is quite important for the RDS, since there are other systems that need its output to do their own tasks: user localization and identification, voice activity identification, arousal level detection, *etc*.

**Figure 4.**
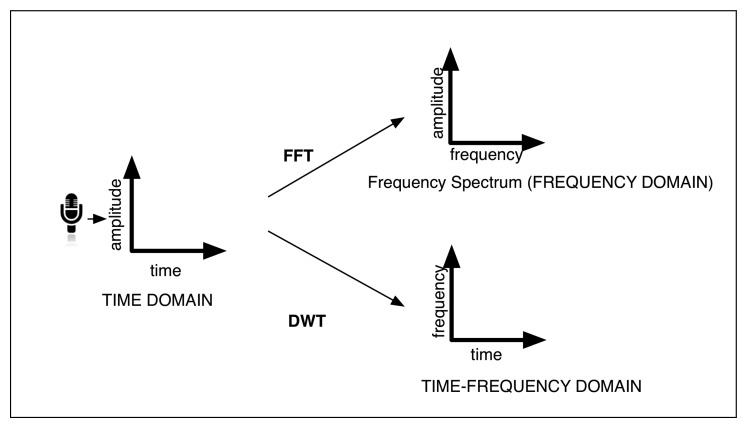
The three audio domains in which voice feature extraction is performed.

This voice features extraction module is being continuously executed, while the user voice is being perceived. The extracted features, called statistics, are obtained in the time domain or in the frequency domain using the Fast Fourier Transform (FFT); or in the time-frequency domain using the Discrete Haar Wavelet Transform (DWT) [59] (see Figure 4). These statistics are the following:

- Pitch
- Flux
- RollOff
- Centroid
- Zero-crossing rate (ZCR)
- Root Mean Square (RMS) (Volume)
- Signal-to-noise ratio (SNR)
- Communicative rhythm.

Once a user stops talking, the module takes into account the maximum, minimum and average values of the statistics described. Hence, we have the final set of statistics: *pitch FFT average, pitch FFT min, pitchFFT max, pitch FFT amplitude, pitch autocorrelation average, pitch autocorrelation min, pitch autocorrelation max, pitch autocorrelation amplitude, pitch DWT average, pitch DWT min, pitch DWT max, pitch DWT amplitude, centroid average, centroid min, centroid max, centroid amplitude, rollOff average, rollOff min,rollOff max, rollOff amplitude, gain medium, flux medium, flux min, flux max, flux amplitude, volume max, volume mean, SNR average, SNR max, ZCR average, ZCR min, ZCR max, ZCR amplitude and communicative rhythm*.

1. **Pitch**: This statistic refers to the frequency of the sound perceived by the human ear. It depends on the number of vibrations produced by the vocal chords. The pitch calculation is not included among the components of Chuck, so it has been implemented by the authors. In this work, we have developed three different Pitch Detection Algorithms (PDA) working in distinct domains:
  (a) The first method detects the first minimum in the autocorrelation of the input signal in the time domain. That is, the pitch corresponds to the minimum in the cross-relation of the audio signal with itself [60,61].
  (b) The second method applies the FFT, so it works in the frequency domain. This algorithm considers the distance among peaks in the spectrum of the signal to calculate the pitch.
  (c) The last method to obtain the pitch works in the time-frequency domain, and it uses the Discrete Haar-Wavelet Transform (DWT). It is based on Larson's work [62].
2. **Flux** indicates if there are big variations in the frequency domain. Values near zero indicate that the obtained amplitude values, in all frequency ranges, are very similar. Values near one indicate that there are important variations in the frequency domain, showing a very sharp spectrum. Statistic flux tells us if the signal is concentrated around an average amplitude or if there are many variations in the volume of the signal.
3. **Rolloff-95** corresponds to the frequency value at which 95% of the signal energy is already contained. For example, if the signal is contained between 200 and 3,000 Hz, maybe, at a frequency of 2,800, the amount of energy contained within the range of zero to 2,800 is already 95% of the total. Then, the RollOff-95 value is 2800.
4. **Centroid** represents the median of the signal spectrum. That is, the frequency the signal approaches the most. It is frequently used to calculate the tone of a sound or voice (timber). Our vocal apparatus produces very different values to those produced by a violin or a flute.
5. **Zero-crossing rate** indicates the number of times that the signal crosses the zero (x axis). Typically, the ground noise crosses much more times the x axis than a verbal sound.
6. **Signal-to-noise ratio (SNR)** allows us to relate the voice signal volume (RMS) to the noise signal volume. In order to do that, the average volume value of the noise when there is no voice activity is calculated. Besides, the average volume value of the received signal, when the voice activity is detected, is also calculated. Finally, SNR is obtained by dividing the first obtained value by the second one. Therefore, this statistic allows, in noisy environments, for differentiating the user voice from the noise better than just using RMS.
7. **The communicative rhythm** serves to obtain more information about the state of the user. This parameter is obtained by counting the number of words pronounced per minute. Each word is separated from the previous one by a brief instant of silence or a pitch peak, which are identified. This sum of silences and pitch-peaks is multiplied by 60, and this result is divided by the duration (in seconds) of the speech. This statistic is very useful to distinguish emotions, because each of them have typically different communication rhythms.

### 4.2. Classifier

Once the voice features have been extracted, it is necessary to classify them into emotions, that is, to decide which emotion corresponds to which values. Next, three possible solutions to train the classifier are shown.

- **Offline:** this approximation consists in training the automatic emotion detection system before its use in the RDS. There are, at least, two possibilities:
  1. A unique universal classifier (for all types of users) previously trained using examples of sentences from a great variety of users (different ages, languages and gender). Nevertheless, in the literature, there are some works that state that using a classifier for each user is more precise than using a universal one [53,63].
  2. A classifier for each possible language: It is assumed that this option would obtain greater success rates. The RDS would inform the emotion detection module about the language of the user. Another variation would be the use of two different classifiers for each language: one for masculine and one for feminine voices.
- **Online:** This option implies building the classifier during real interactions with the user using the RDS. During the registration phase (which is carried out only once per user) the system learns the characteristics of the user, such as his name, language, voice tone, the way he expresses his emotions, *etc*. Two possibilities are considered to build the classifier during the registration phase:
  1. The user registers in the system, and an external supervisor tags each locution with the perceived emotion. This approach presents several disadvantages: first, the user, during the registration phase, will probably not express the four selected emotions; second, although the user expresses all of them, he/she will not do it many times, so only a very small amount of examples would be obtained for each emotion; and third, we need a supervisor to tag such emotions.
  2. During the registration phase, the system asks the user to express each emotion it needs to learn. Therefore, the user must simulate these emotions multiple times to facilitate the learning of features, and the system builds the classifier. In this case, the dialog is the supervisor, not a person. The disadvantages are related to the fact that the faked emotions are not always so similar to the natural ones. Besides, this registration process should be similar to the first moment of meeting among humans, and “faking” emotions does not seem to be very natural.
- **Mixed:** in this approximation, a classifier is built based on the relative relationships among the extracted features instead of their absolute values (e.g., happiness is related to a pitch 20% higher than the neutral emotion). The construction of this classifier would be *offline*. Later, during the registration phase, in real (*online*) interactions, the statistics values associated with the neutral tone of the user are obtained. Using these values, the classifier is scaled offline, such that these relative relationships among features are converted into rules (as absolute numeric values). This approach requires the assumption of the idea that the user emotion, during the registration phase, is neutral (this approach is proposed in [63]). The main disadvantage of this approximation is that it will not work properly if the user registers in the system using a non-neutral tone voice.

In this work, the selected option is the building of a universal emotion classifier trained *offline*. For this option, as previously explained, a set of tagged locutions for each emotion (happiness, sadness, surprise and neutral) is needed. The following sources, which provide voice samples related to several emotions in different languages, ages and gender, are used:

- Voice examples from the developers simulating emotions.
- Interviews with colleagues asked to fake emotions.
- Real or spontaneous interactions between the robot and colleagues.
- Interviews obtained from the Internet.
- TV shows from the Internet.
- Audiobooks from the Internet.
- Databases with a tagged voice corpus:
  – Emotional Prosody Speech Database (LDC): with 15 types of emotions.
  – Berlin Emotional Speech Database (EmoDB): with seven types of emotions. See [64].
  – Danish Emotional Speech Corpus (HUMANAINE): with five types of emotions. See [65].
  – FAU Aibo Emotion Corpus: 8.9 h of spontaneous voice recordings from 51 children, with 11 types of emotions. See [66,67].

The automatic learning program, Weka, provides many automatic learning techniques, which allow us to classify from the training patterns. Using this linguistic corpus and our voice features extraction module, a file with training patterns for Weka [68] has been built. This file has about 500 emotion-tagged locutions (these samples are in *arff* format, which is valid for Weka).

During the training phase, the different techniques provided by Weka have obtained the following success rates:

1. **Bayesian**: Bayesian network: 68.95%; Naive Bayes: 65.52%.
2. **Fuzzy logic**. IBK (K-nearest neighbours classifier): 85.65%; IB1: 82.22%; LWL(Locally weighted learning): 62.74%.
3. **Rules**: JRIP (Routing Information Protocol): 81.15%; ConjuntiveRule: 61.88%; DecisionTable: 70.02%; ZeroR (determines the most common class): 57.17%; PART (a partial C4.5 decision tree): 77.94%.
4. **Decision tree learning**: J48 (a pruned or unpruned C4.5 decision tree): 80.51%; BFTree (a best-first decision tree classifier): 79.01%; LADTree (a multi-class alternating decision tree using the LogitBoost strategy): 68.95%; LMT (classification trees with logistic regression functions at the leaves): 79.44%.

The final selection of the classifiers integrated in GEVA is based on the best results obtained using the “cross-validation” method over the training set. Besides, the simplicity of their implementation using Chuck has also been considered. The selected algorithms are the following:

- Decision tree learning J48: This is an implementation of the tree decision C4.5 made by Weka, used in data mining or automatic learning.
- Decision rule JRIP. The Routing Information Protocol is a vector-distance algorithm used in data mining or automatic learning.

Using these classifiers, GEVA gives the emotion and gender outputs and their confidence values. Moreover, a novel *voice activity detection* mechanism is implemented in GEVA. This mechanism is able to determine the start and the end of the voice locutions. In general, the majority of the systems that work with voice signals detect these moments based on a volume threshold. Nevertheless, this approach (implemented in the first version of GEVA) is not robust, since it is not able to differentiate between the human voice and other kinds of sounds or noises. The new developed mechanism for human voice detection is more complete, since it considers a bigger number of statistics.

## 5. The Emotion Detection Visual-Based Component: GEFA

In many other works, the visual information, especially the one related to the user's face, is used alone [69] or together with the voice [25,38]. In [70], an extensive review of different techniques and research related to the visual emotion detection process is presented. The steps followed in this process are similar to the ones previously introduced for the emotion detection by voice:

- Face detection: to detect the user face in the image flow.
- Facial features extraction: features, such as, eyes distance, mouth shape, *etc*.
- Emotion classification: from the extracted features, the classifier is built.

### 5.1. Face Detection

The user face detection and its tracking have been extensively studied. There are some works, such as the one presented by Viola in 2004 [71], which describe the face detection process using robust automatic systems. Besides, the well-known artificial vision library, OpenCV, also has the needed functions to do this task [72].

### 5.2. Facial Features Extraction

Once the user face has been detected in the observed scene, the next step is to extract the information about the facial expression in an automatic way. There are several computational approaches to represent and extract the information associated with that facial expression.

One of them is based on interest points, also known as “local approximation”, and the geometric relationships among them [73,74]. In this approach, the detection of certain points of the face is needed. Using these points and their relationships, the geometric features are obtained.

Another approach is known as the “holistic approach”, since the face is represented as a whole unit. In this approach, a 3D mesh is situated over the detected face [75–77]. This mesh, also known as “active appearance model”, uses the difference between the current estimate of appearance and the target image to drive an optimization process. This approximation presents more robustness regarding the head movement in real time and to partial occlusions and illumination changes, in comparison with the local approximation.

Finally, the face can be modeled using a hybrid approach that combines the holistic approach and the interest points (the local approximation). In this approach, the interest points are used to determine the initial position of the mesh [78].

The facial feature extraction must be made without loosing information. Nevertheless, some factors, such as hair, glasses, *etc.*, complicate this task, since they can hide some parts of the face. Another problem is the size and orientation of the face in the input images. Furthermore, the noise in the images is another inconvenience.

### 5.3. Facial Expression Classification

As already stated, the next step is to analyze the facial expression. Although the human mechanism for face detection is very robust, the one for the facial expression detection does not work so well. A trained observer is able to detect facial expressions correctly just 87% of the time, and this number varies according to the familiarity of the observer with the interlocutor [79].

The Facial Action Coding System (FACS) [80] is probably the best known study about facial activity, although there are other similar coding schemes, such as EMFACS (Emotional FACS [81]), MAX (The Maximally Discriminative Facial Movement Coding System) [82] and AFFEX (a system for Identifying Affect Expressions by Holistic Judgments) [83]. They constitute a representation developed to help psychologists to code facial expressions from static images. FACS is based on the enumeration of all the “action unities” (AU) of the face, 46 more precisely, that can cause facial movements. The combination of these AUs generates a big set of possible facial expressions. Nevertheless, a well-known limitation of FACS is that this system lacks detailed temporal and spatial information at a local and global scale [84].

There are some studies [32,85] about the possibility of having a universal classification of facial expressions in emotions that takes into account the great variability among people, their gender, age, culture, *etc*. Moreover, it must be considered that emotions can be expressed with different intensities, and in cases of low intensity, the differences among facial expressions are minimal. However, if the intensity is high, the differences are more easily detected.

Finally, there are some psychological studies that argue that the temporal factor of facial expressions is critical for their interpretation [79,86,87]. For this reason, these researchers have decided to use real-time systems, which analyze the face as a whole, and where each AU can be inferred from the previously detected ones.

### 5.4. Complete Solutions That Integrate Face Detection, Feature Extraction and Emotion Classification

There are several works that present the three phases needed to detect emotions visually (face detection, features extraction and emotion classification) [76,88]. They obtain improvements in their results thanks to the use of automatic learning techniques and databases with a large number of samples. Nevertheless, they do not give any information about source codes,or user libraries to check their results.

**Figure 5.**
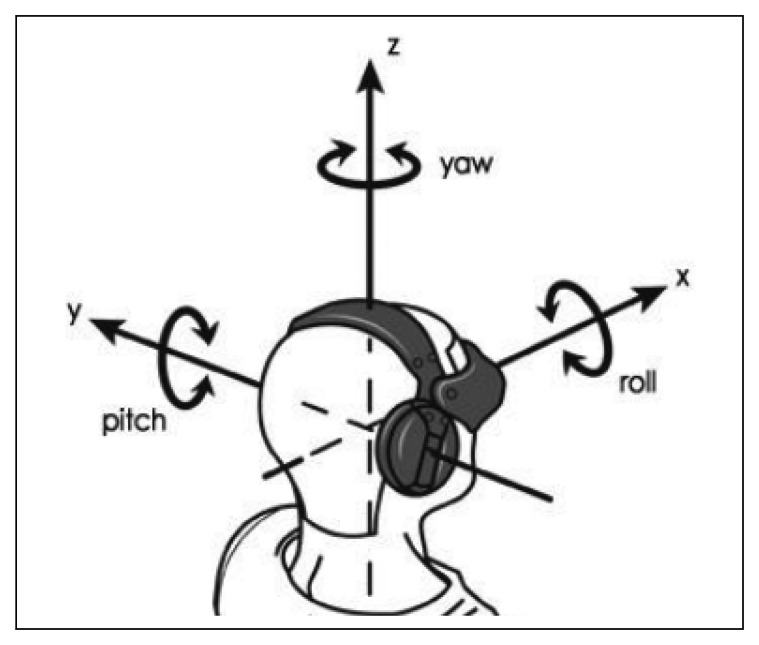
Rotation parameters: roll, pitch and yaw.

In a recent work (2011), Littlewort [89] presents a visual tool called CERT [90], which allows emotion classification in real-time (six emotions) and the detection of 19 FACS AUs. For the feature extraction, this tool uses the approach based on interest points (local approximation), and for the classification of each possible AU, it uses a Support Vector Machine (SVM) algorithm. The developers claim success rates between 80% and 90.1% for the emotion recognition online. Moreover, this tool is provided with a head-pose detector for emotion recognition in different poses (see Figure 5). The valid range for *roll* (tilting side to side or turning on the imaginary axis that connects the nape with his nose) is −9° to 9°, for *pitch* (tilting forward and backward or turning on the imaginary axis that connects both ears) is −8° to 15° and for *yaw* (turning left and right or turning on the imaginary axis that connects the neck with the crown) is −25° to 25°.

Another interesting work is the one presented by the Fraunhofer Institute for Integrated Circuits (Erlangen, Germany), where the library, sophisticated high-speed object recognition engine (SHORE), is presented [91,92,94]. This software package is able to detect faces with low CPU consumption and robustness regarding illumination changes. Moreover, it is able to track the position and orientation of users faces. In order to do this, it implements the following three face model types (holistic approach): *front, rotated* and *profile*. The *front model* is for interaction with roll, pitch and yaw values close to “zero”. The *rotated model* is for interaction with roll values between −60° to 60° and, again, with pitch and yaw close to “zero”. Finally, the *profile model* is for interaction with pitch and yaw values not close to “zero”. The detection rate in the *front type* is much higher than the ones of the other models, and the false detection rate is respectively lower. Apart from the user emotion detection, SHORE is able to identify users and to detect gender and age. A demo of the software can be downloaded from [93].

In our work, after reviewing the literature and testing several available libraries, the presented CERT and SHORE tools have been selected to build the GEFA module. Both of them have been integrated in our system as Robotic Operative System (ROS) nodes that use the ROS communication facilities. Due to the limitations of CERT and SHORE, the interaction system (RDS) should always try to place the robot in front of the user in order to improve the success rates.

We have observed that the face detection and the emotion classification of CERT work up to a 2 m distance between the camera and the user; within this range, there are no differences in the the classification accuracy. However, if the interaction distance is bigger than 2 m, the face is lost, and the system does not work properly. This is independent of the resolution of the camera used, since this happens in the same way when using low resolution (1.3 megapixels) and high resolution cameras (15 megapixels). In the case of the software package, SHORE, the maximum interaction distance is about 4.5 m. Again, the success rate, within this distance, is not affected by the interaction distance or the resolution of the camera.

## 6. Integration of GEVA and GEFA

The final multimodal emotion detection system is the result of the concurrent integration of two components:

1. GEVA: this is the module in charge of extracting the voice features and of classifying emotions and gender. As previously explained, this module is written in the Chuck language, and it can be executed in Ubuntu, as well as in a Mac. Although it is multi-platform, there are several factors that make the implementation difficult: the implementation in Chuck, the differences between the audio systems of both OSs (CoreAudio in MacOS and Advanced Linux Sound Architecture, called ALSA, with portaudio in Ubuntu), the kind of microphone used, *etc*.. This module analyzes the human voice and gives as outputs the user gender and one of the four possible emotional states: happiness, sadness, surprise and neutral, independently of the user and his language.
2. GEFA is formed by:
  - the third-party *SHORE* for the facial expression recognition. This package gives the intensity values of the following emotional states: happiness, sadness, surprise and anger (as already mentioned in Section 3.1, this last emotion is not considered in our system). Moreover, if none of these emotional states has an intensity greater than 50%, then, it is assumed that the emotional state of the user is neutral. On the other hand, this package also gives the approximate user age with a margin of error in its estimation and the user gender (with no confidence value).
  - The third-party *CERT* is also used for facial expression recognition using artificial vision. This package gives the intensity value for each detected emotion. In this case, the outputs are: fun, joy, smile detector (these three are grouped in one set as happiness), disgust and sadness (grouped as sadness), surprise, neutral, fear and anger (these last two are not considered in our system, as mentioned in Section 3.1). Moreover, the user gender is also obtained.

The association of similar emotions in just one emotion is made to unify the outputs of both modules. For this reason, it is easier to establish a rule to specify how to combine the output of each module to determine the principal emotion of the user.

The final goal is to detect the main emotion of the user and to integrate this information in the interaction system RDS. This integration is made by adding to each CA the emotional state of the user (if the detection is possible), so that, as already introduced in Section 1, the information included in the CA is the semantic values associated with the transmitted sentence, the name of the user, the localization in relation to the robot, the pose of the user body, the touched part of the robot body and the detected user emotion.

The proposed multimodal emotion detection system is integrated in the robot as follows: each component, GEVA and GEFA, is integrated in our control architecture (AD-ROS, an extension of the ROS architecture) as nodes and emit messages (called topics) communicating the detected emotions. Each classifier of GEVA has an associated ROS message, as well as CERT and SHORE (GEFA). The component that applies the decision rule, that fuses these outputs in one, is prescribed to these messages and to the one that informs of the beginning and the end of the user vocalization. This component uses this information and applies the decision rule. Finally, it emits a message with the main user emotion.

### 6.1. Decision Rule

**Figure 6.**
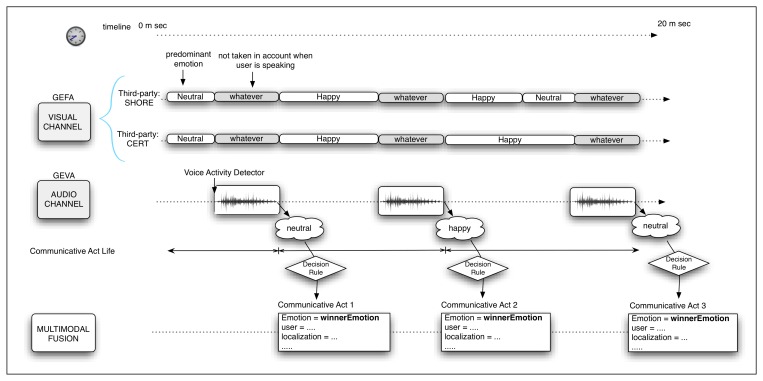
Scheme of the process for determining the main user emotion in each communicative act (CA).

In Figure 6, the process to determine the main user emotion during the CA can be observed. It has been considered that the visual emotion detection system does not work properly when the user is talking. Therefore, when the user is quiet, just the output of the GEFA (visual) module is considered, and in the other case, only the GEVA module, based on the user voice, is taken into account. Once the vocal activity finishes, the CA also ends. Considering this temporal constraint, it is necessary to define a decision rule to combine the information given by each module to generate a single output. The final definition of this rule depends on the success rate (accuracy) of each individual module in relation to each emotion. Hence, the decision rule is settled after the results of the experiments of each module, GEVA and GEVA, are obtained.

Once the success rate of each module (with two classifiers each) is estimated, then the decision rule is defined. In order to do that, it is necessary to determine the confidence degree of each one of the outputs (the detected emotion) of the classifiers.

In this work, the *confidence degree* of each output is calculated using the Bayes theorem and the confusion matrices. The Bayes theorem is applied as follows:

Let us assume that the current state of the system is *S* and that the list of states is finite: S = {*s*_1_,…, *s_n_*} where *n* ∈ ℝ.

Then, let us suppose that there is a classifier, *C*, the output of which is *S_C_* ∈ **S**, which is used to estimate *S* ∈ **S**.

The *confusion matrix*, *M* ∈ ℕ*^n^*^×^*^n^*, of the classifier, *C*, is defined as follows:

(1) $$\forall i,j\in [1,n],M_{ij}=p(S_{C}=s_{j}|S=s_{i})$$

where *s_C_* is the state estimated by the classifier, *C*.

As such, the value in row *i* and column *j* corresponds to the probability that the classifier estimates the state *S_C_* = *s_j_* given the real state *S* = *s_i_*.

Therefore, an ideal classifier would have a diagonal confusion matrix, so *M_ij_* ≠ 0 if *i* = *j* and *M_ij_* = 0, if *i* ≠ *j*. That is, the detected states are always equal to the real ones.

In order to determine the confidence degree of the classifier, *C*, we need to estimate the probability of the system being in a real state, *s_i_*, when the classifier output is *s_j_*. In other words, we want to compute *p*(*S* = *s_i_*|*S_C_* = *s_j_*).

(2) p(S=si|SC=sj)=(Bayes'theorem)p(SC=sj|S=si)p(S=si)p(SC=sj)

(3) $$\begin{array}{ll} = & \frac{p(S=s_{i})p(S_{C}=s_{j}|S=s_{i})}{\sum_{k\in [1,n]}p(S_{C}=s_{j}\cap S=s_{k})} \end{array}$$

(4) $$\begin{array}{ll} \underset{\left(\text{Kolmogorov definition}\right)}{=} & \frac{p(S=s_{i})p(S_{C}=s_{j}|S=s_{i})}{\sum_{k\in [1,n]}p(S_{C}=s_{j}|S=s_{k})p(S=s_{k})} \end{array}$$

(5) $$\begin{array}{ll} = & \frac{p(S=s_{i})M_{ij}}{\sum_{k\in [1,n]}M_{kj}p(S=s_{k})} \end{array}$$

In the case where all states are equally probable, the probability of *s_i_* given *s_j_* is:

(6) $$p(S=s_{i}|S_{C}=s_{j})=\frac{M_{ij}}{\sum_{k\in [1,n]}M_{kj}}$$

Let us now suppose we have a collection of *m* classifiers **C** = *C*_1_,…, *C_m_*, *m* ∈ ℝ.

We want to estimate *S*, the current state of the system. We know the output of each classifier, *S_C_* ∈ **S**, ∀*C* ∈ **C**.

As seen, for each possible state of the system, *s_i_* ∈ **S**, and for each classifier, *C* ∈ **C**, we can estimate *p*(*S* = *s_i_*|*S_C_*).

**Decision rule:** knowing all these conditional probabilities, an easy-to-estimate state is the state, *ŝ*, that has the highest probability among all classifiers:

(7) $$\hat{s}\in S,\hat{C}\in C|\forall s_{i}\in S,\forall C\in C,p(S=s_{i}|S_{C})\leq p(S=\hat{s}|S_{\hat{C}})$$

## 7. Estimating the Confusion Matrices of GEVA and GEFA

This section describes the experiments performed to determine the accuracy of GEVA and GEFA separately (without applying the decision rule, which determines the final predominant user emotion). As previously explained, these experiments are necessary to apply the decision rule that joins both outputs in a single final emotion.

### 7.1. Experimental Setup

The experiments made to test the performance and to obtain the confusion matrices of GEVA and GEFA were carried out in several sessions at the *Robotic center ISR* (*Institute for Systems and Robotics*) *in Lisbon* (Portugal).

**Figure 7.**
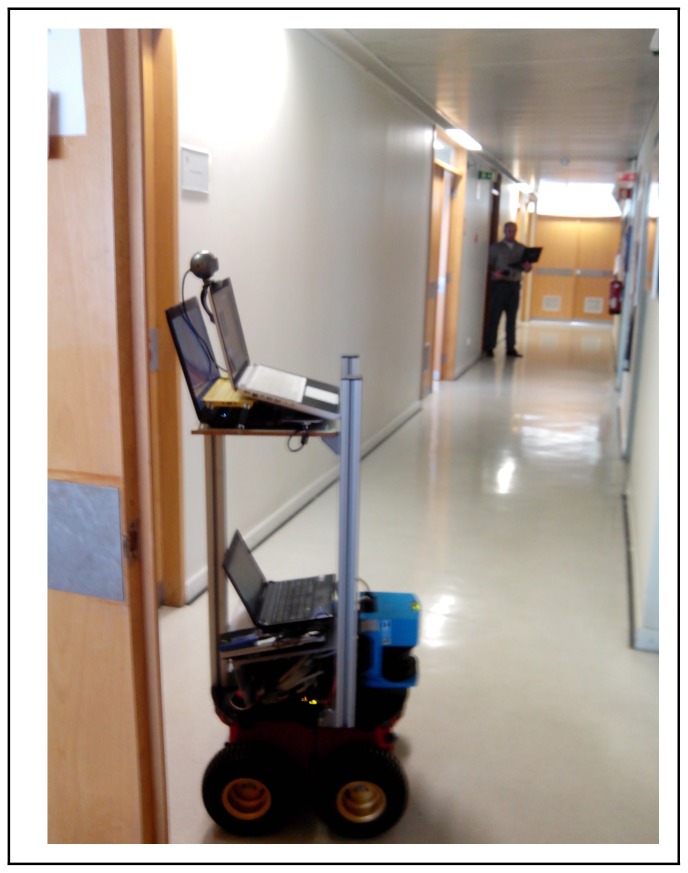
The robot used in the experiments.

These experiments were made using a Pioneer robot (Figure 7). The configuration used is the following:

- An Apple Macbook with MacOS executing CERT and GEVA. This computer integrates a camera *iSight* of 1.3 megapixels and an omnidirectional microphone with noise cancellation. The output, visual and audio, was recorded using Quicktime for its offline analysis.
- A portable computer with Windows 8 executing SHORE with an external webcam of 2 megapixels. The output was also recorded (with no audio).
- A netbook with Ubuntu 12.04 executing a non-verbal sounds generation program [95]. This computer is not relevant for the analysis of the experimental results, but it has been useful for catching the user attention.

**Figure 8.**
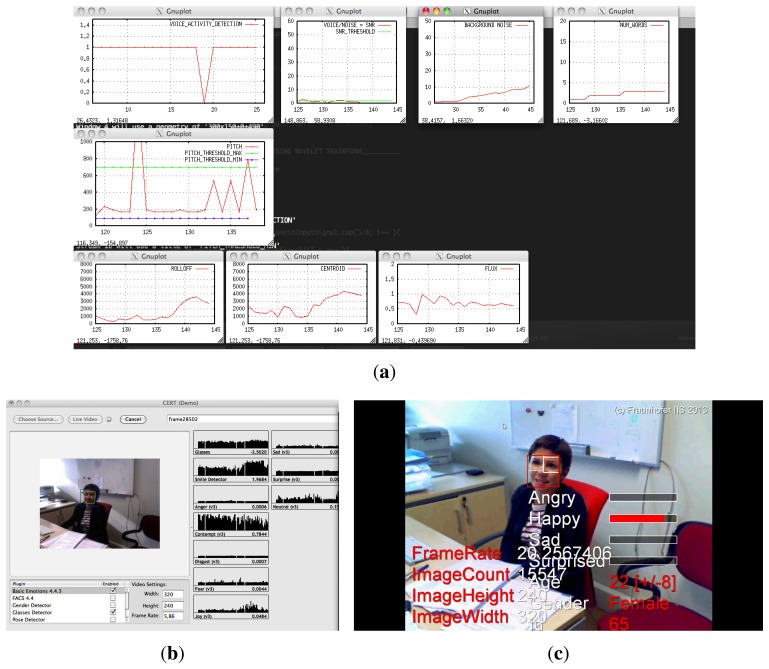
Image taken during the experiments carried out in the ISTin Lisbon. (**a**) Gender and Emotion Voice Analysis (GEVA); (**b**) Computer Expression Recognition Toolbox (CERT); (**c**) Sophisticated High-speed Object Recognition Engine (SHORE).

A screen shot of the experiment recordings given by the portable Mac and Windows PC can be observed in Figure 8. This figure shows the graphical interfaces of GEVA, CERT and SHORE. In these online graphical outputs, several parameters (such as the statistics, the detected emotions, *etc.*) are plotted.

In the experiments performed at the IST/ISR, the robot first moved freely around the environment and approached possible users. In total, ten users interacted with the robot in a natural way. The robot tried to catch the user's attention by emitting non-verbal sounds (a special kind of “robotic” language). The goal of this first part of the experiment is to engage the users with the robot and to detect their spontaneous emotions. In this case, the robot was tele-operated by supervisors. During these interactions, the users tended to situate themselves in front of the robot. When the user talked to the robot, it responded to the user using non-verbal sounds that tried to imitate the user voice. The content of the user speech is not important for the system, since the features extracted from the user voice are not related to the content of his speech. Moreover, in these experiments, there is no script or previous indications; the users, moved by their curiosity, approached the robot and tried to interact with it.

The main observed problem of this first approach was that not all the possible emotions were expressed by the users (basically, the users only expressed neutrality and happiness during the HRI).

Therefore, due to this limitation, a second experiment, with 40 undergraduate students (80% male and 20% female) of different nationalities was carried out. The experiment was performed in a classroom in which each student participated separately. During the interaction, the user is in front of the robot and one meter away from it. In this experiment, the users were asked to fake a certain emotion (by voice and expressing it through facial expression) for 3 s at least (enough time to detect the user emotion). Each student was asked to fake the four emotions. Moreover, they were asked to try to do it in the most natural way (not very overacted).

Later, a similar experiment was made during a seminar for PhD and postgraduate students, around 20 people (only one woman). The test was performed in a small classroom. Again, the system was tested with each of them, and they were asked to face the robot and to fake the four emotions. During all these interactions, the distance between the user and the robot was not greater than 2 m.

All these experiments were recorded: the outputs (the emotions detected by each component) and the inputs (the visual and audio signals). In the case of natural interactions, the supervisor decides if the detected emotion is correct. Meanwhile, in the case of the faked emotion, the verification is straightforward.

### 7.2. GEVA Confusion Matrices

From the audio data obtained during the experiments, with natural and faked emotions, two confusion matrices have been obtained: one for each classifier used (J48 and JRIP). The rows show the real emotions expressed by users (natural and faked), and the columns show the detected user emotion by the classifier. The sum of the values of the entire row must be equal to 100.

In Table 1, it can be observed that the classifier, J48, is not very accurate at distinguishing between the tone of voice for happiness and neutral when happiness is the real expressed emotion. Nevertheless, this same classifier is quite precise (80.28%), recognizing the neutral emotion of the user. On the other hand, when the user is sad, the classifier is able to detect this emotion 66.66% of the time, and the rest of the time, it incorrectly detects that the expressed emotion is neutral. Finally, J48 has problems detecting the surprise emotion successfully. In this case, the success rate is just 28.57%, although it can be observed that it never gives a false positive when detecting this emotion. The J48 just gives the surprise output when the user expresses this same emotion.

In order to calculate the average success of the classifiers, it is necessary to sum the elements of the diagonal and divide this result by the number of emotional states (4). *Therefore, for the classifier, J48, the average success rate is 56.37%*.

In relation to the other classifier, the JRIP, it can be observed that it is quite accurate at detecting the neutral and sad emotions. On the contrary, it has problems recognizing happiness and surprise. *Finally, the average success rate in JRIP is 57.10%*.

Although these matrices have been calculated using the natural and the faked interaction, it has been observed that the failure rate significantly increases when the user tries to simulate the emotion asked by the supervisor and the user does not do it in a very expressive way. In these cases, even the supervisor had problems recognizing the user emotion, and the classifiers give more incorrect answers. For example, the happiness or sadness emotions expressed in a timid way may be confused with the neutral one. In the same way, if the user expresses the neutral emotion in a very relaxed way, it can be confused with sadness.

### 7.3. GEFA Confusion Matrices

As previously explained, GEFA is formed by two third-party software packages: SHORE and CERT. As already said, using the recorded visual information obtained from the experiments, the confusion matrices for both packages are calculated and shown in Table 2.

Again, it can be observed that SHORE is quite precise at detecting the happiness (100%) and the surprise (80%) emotions. On the contrary, it does not work so well when detecting the neutral (66%) and sad (55.55%) ones, although these values are not really poor. In this case, *the calculated average success rate for SHORE is 75.55%*.

In relation to CERT, as can be observed, this package is very accurate at recognizing happiness (100%) and the neutral (85.71%) emotions and not so precise when detecting sadness (28.57%) and surprise (36.36%). Finally, *the calculated average success rate for CERT is 62.66%*.

As mentioned in Section 6.1, during the experiments, we observed that the considered software packages are not reliable when the user is speaking. When this happens, the outputs of these packages fluctuate between different emotions.

## 8. Experiment with the Multimodal User Emotion Detection System

As explained in Section 6.1, once the confusion matrices of both components (GEVA and GEFA) have been experimentally obtained, the decision rule can be applied to determine the main user emotion. In order to understand this decision rule, let us apply it to the following example: the user greets the robot, and it perceives the voice and face of the user. Let us assume that the real user emotion is sadness.

In our approach, the states and classifiers are the following:

**S** = {*happiness, sadness, neutral, surprise*}
**C** = *J*48, *JRIP, CERT, SHORE*

Let us assume that the robot, according to the values of the confusion matrices, receives the outputs of each classifier:

1. J48 (GEVA) → Neutral (estimated emotion)
2. JRIP (GEVA) → Sad (estimated emotion)
3. CERT (GEFA) → Neutral (estimated emotion)
4. SHORE (GEFA) → Neutral (estimated emotion)

Applying the decision rule defined in Equation (7), the following results are obtained (in this experiment, it is assumed that all emotions are equally probable. This is the case, since the users are asked to simulate each of them):

(8) $$p({\text{Neutral}}_{\text{real}}|{\text{Neutral}}_{J48})=\frac{80.28}{50+80.28+33.33+42.85}+0.38$$

(9) $$p({\text{Sad}}_{\text{real}}|{\text{Sad}}_{\text{JRIP}})=\frac{77.77}{0+12.67+77.77+0}=0.82$$

(10) $$p({\text{Neutral}}_{\text{real}}|{\text{Neutral}}_{\text{CERT}})=\frac{85.71}{0+85.71+71.42+54.54}=0.40$$

(11) $$p({\text{Neutral}}_{\text{real}}|{\text{Neutral}}_{\text{SHORE}})=\frac{66.66}{0+66.66+22.22+10}=0.67$$

According to these results (the results shown are the highest values given by each classifier), the classifier with the biggest confidence in its estimation is the JRIP used by GEVA. This may seem to be contradictory, since the other three classifiers estimate that the emotion expressed by the user is neutral instead of sad. Nevertheless, the decision rule decides that the output given by the classifier with the biggest confidence is the one that must be considered. In the case that the winner classifier does not have a sufficiently high confidence (under a certain threshold), then it can be determined that the estimation is not reliable. Therefore, in this case, the information about the user emotion cannot be obtained.

If this decision limit is low, there is a risk of obtaining false positives (accepting emotions that have not been expressed by the user). On the contrary, if the limit is very high, then the dialog system will frequently lack information about the user emotion.

### 8.1. Performance of the Multimodal User Emotion Detection System Statistically Computed

As already said, the confusion matrices for each classifier have been computed. Since these matrices give us the probability of detecting a certain emotion given a real user emotion, one way to test the performance of the whole system would be the following: first, to generate a big number of examples (real emotions as inputs) and, for each of them, to calculate the outputs of each classifier (according to their confusion matrices); second, as in the previous example, to apply the decision rule selecting the final detected user emotion with the highest confidence value.

To obtain the statistically-computed confusion matrix of the multimodal user emotion detection system, we have generated 10,000 examples by computer. These examples were randomly generated, and each emotion has the same probability of being selected (25%). Checking the right and the wrong emotion detection, the values of the confusion matrix are obtained. These results are shown in Table 3.

*The calculated average success rate of the whole system is 83%*. This value confirms that the use of the information from the audio and visual channel and the application of a decision rule improve the average success rate of each classifier: 56.37% for J48, 57.10% for JRIP, 75.55% for SHORE and 62.66% for CERT.

### 8.2. Performance of the Multimodal User Emotion Detection System Tested with Real Users

#### 8.2.1. Experimental Setup

**Figure 9.**
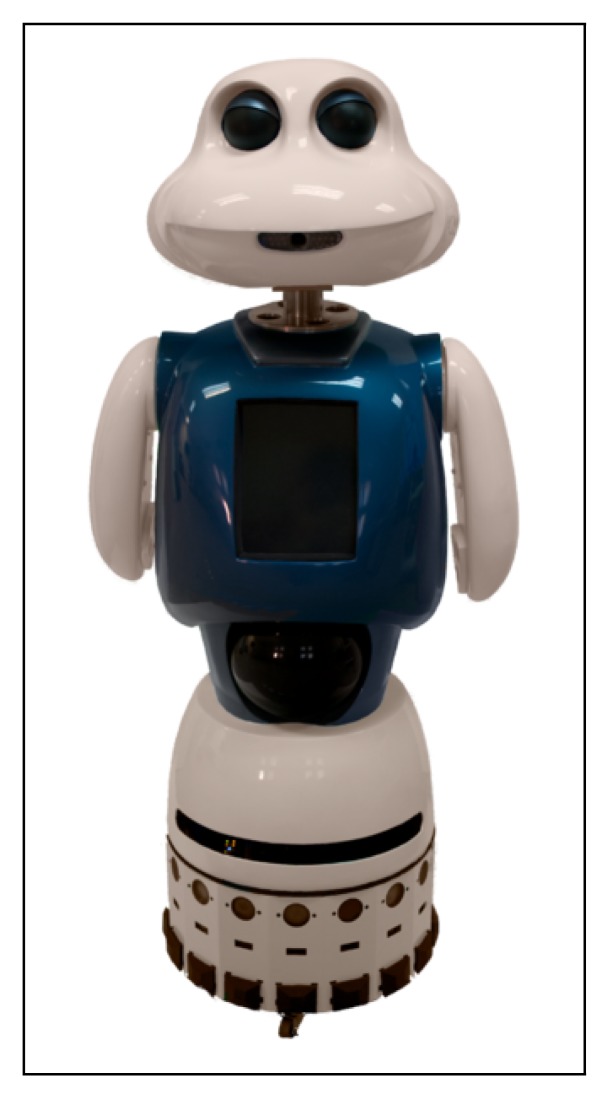
The robot, Maggie.

This last experiment with real users was carried out at the Carlos III University of Madrid (Madrid, Spain), using the robot, Maggie [96] (Figure 9). The complete system has been implemented in its control architecture (AD-ROS) and in its general interaction system (RDS), as explained in Section 6.

A total of 16 users have interacted with the robot as follows: The user enters the lab where Maggie is placed. After that, the robot detects the user and is able to situate itself in front of the user at about 1.5 m away. Then, the robot explains the methodology of the experiment to the user and asks him to express one emotion at a time (visually and by voice) for at least 3 s. The robot also insists on being natural while expressing the emotion. Once the user has expressed the required emotion, the robot asks the user to express a new emotion. Again, all these data, the audio and the visual signals are recorded.

#### 8.2.2. Confusion Matrix of the Multimodal User Emotion Detection System

Again, analyzing the data recorded, the confusion matrix is obtained to prove the usefulness of the whole system; see Table 4. As observed, the biggest values for each row are situated at the diagonal of the matrix, proving the good performance of the system. In this case, when the system is tested with real users, *the average success rate is 77%*. This value is near to the average success rate computed statistically (83%) and better than the ones obtained for each classifier: 56.37% for J48, 57.10% for JRIP, 75.55% for SHORE and 62.66% for CERT.

These results prove that the use of several modes (audio and visual) to detect the user emotion improves the success rate obtained by each mode separately. Moreover, the whole system seems to be sufficiently good at detecting the user emotion. In fact, it does not seem to present any serious problems in recognizing any emotion in particular. In detail, the proposed multimodal system is quite good at detecting happiness, relatively good at surprise and, finally, just fair at recognizing the neutral and sad emotions (although they do present acceptable success rates).

## 9. Conclusions

In this paper a multimodal and automatic emotion detection system applied to the HRI has been described. This system has been implemented in a general interaction (or dialog) system, called RDS. Using the emotion detection system, the user emotion can be included in the information exchanged between the user and the robot during a dialog turn, known as a communicative act (CA). The inclusion of this emotional information will help to increase the adaptation and naturalness of the dialog. The detected emotions are: happiness, sadness, neutral and surprise.

The emotion detection system has been integrated as a module in the RDS, and it has been deeply described. In order to determine the user emotion, two information channels have been used: the audio and the visual ones.

The system, GEVA, is one of the main contributions of this work. This system analyzes the user voice to extract its features using the Fast Fourier Transform and the Wavelet Transform. Once these features have been obtained, two classifiers are used (J48 and JRIP) to determine which emotion corresponds to these features. Moreover, GEVA also has a sophisticated mechanism to determine the beginning and the end of the voice signal, being able to differentiate it from noise.

On the other hand, another module has been developed to detect the emotions through the facial expression analysis: the system, GEFA. This system is formed by two third-party software packages that are able to detect the user emotion using artificial vision: SHORE and CERT.

Several experiments with real users have been carried out to test the performance of both modules separately. From these experiences, a confusion matrix is obtained for each classifier. These matrices give an idea about the success rate of each of them.

The proposed multimodal system fuses the outputs of both modules in order to determine the main user emotion. Therefore, a decision rule is needed. This rule uses the information contained in these matrices to calculate the confidence degree of the output given by each classifier. Moreover, it also considers the particularities of each classifier to determine the right emotion. Another advantage of this rule is that, in the case of adding new information channels with new classifiers, it does not need to be rewritten.

The whole system has been also tested with real users, obtaining a high average success rate. The results prove the benefits of using this multimodal system, instead of considering just one information channel.

As future work, the inclusion of additional information sources, such as the dialog context based on their background, as well as on semantic information of the recognized voice sentence could improve the success rate of the emotion detection.

## Figures and Tables

**Table 1.** Confusion matrices for GEVA (rows: real emotions; columns: detected emotions).

**J48Classifier**						**JRIP Classifier**				
	
	**Happy**	**Neutral**	**Sad**	**Surprise**			**Happy**	**Neutral**	**Sad**	**Surprise**
	
**Happy**	50	50	0	0		**Happy**	30	70	0	0
**Neutral**	0	80.28	19.71	0		**Neutral**	0	87.32	12.67	0
**Sad**	0	33.33	66.66	0		**Sad**	0	22.22	77.77	0
**Surprise**	28.57	42.85	0	28.57		**Surprise**	16.66	50	0	33.33
	

**Table 2.** Confusion matrices for Gender and Emotion Facial Analysis (rows: real emotions; columns: detected emotions).

			
		**Happy**	**Neutral**	**Sad**	**Surprise**			**Happy**	**Neutral**	**Sad**	**Surprise**
			
**SHORE**	**Happy**	100	0	0	0	**CERT**	**Happy**	100	0	0	0
	**Neutral**	0	66.66	16.66	16.66		**Neutral**	0	85.71	14.28	0
	**Sad**	0	22.22	55.55	22.22		**Sad**	0	71.42	28.57	0
	**Surprise**	0	10	10	80		**Surprise**	9.09	54.54	0	36.36
			

**Table 3.** Statistically-computed confusion matrix (rows: real emotions; columns: detected emotions).

	**Happy**	**Neutral**	**Sad**	**Surprise**
**Happy**	100	0	0	0
**Neutral**	0	46.77	41.46	11.76
**Sad**	0	1.635	96.66	1.701
**Surprise**	4.345	2.639	2.60	90.411

**Table 4.** Experimental confusion matrix (rows: real emotions; columns: detected emotions).

	**Happy**	**Neutral**	**Sad**	**Surprise**
**Happy**	100	0	0	0
**Neutral**	6	67	27	0
**Sad**	15	18.33	66.66	0
**Surprise**	0	12	10	78
